# Epidemiological Study of the Discoid Meniscus: Investigating Demographic-Based Predictors in Large-Scale Claims Database

**DOI:** 10.7759/cureus.20050

**Published:** 2021-11-30

**Authors:** Sahej Randhawa, Emily Tran, Nicole A Segovia, Theodore Ganley, Marc Tompkins, Henry Ellis, Kevin G Shea

**Affiliations:** 1 Orthopaedic Surgery, University of California San Diego School of Medicine, La Jolla, USA; 2 Orthopaedic Surgery, Stanford University School of Medicine, Lucile Packard Children's Hospital, Palo Alto, USA; 3 Orthopaedic Surgery, Children's Hospital of Philadelphia, Philadelphia, USA; 4 Orthopaedic Surgery, University of Minnesota School of Medicine, Minneapolis, USA; 5 Orthopaedic Surgery, Texas Scottish Rite Hospital for Children, Dallas, USA

**Keywords:** optum, incidence, race, sex, age, demographics, epidemiology, discoid meniscus

## Abstract

Purpose

To better define the epidemiology of discoid meniscus by analyzing a large, national database for incidence rates and associations with demographic variables.

Methods

From Optum’s Clinformatics® Data Mart Database, incidence rates and proportions of reported racial categories - Asian, Black, Hispanic, and Caucasian - of diagnosed discoid meniscus cases (n = 198) in the study population of patients receiving arthroscopic meniscectomy or repair procedures (n = 60,042) were calculated and compared via chi-square tests to the total population. To control for age, sex, and socioeconomic factors such as income, multivariable logistic regression analysis was performed.

Results

Proportions of discoid meniscus patients who were Asian, Black, Hispanic, or Caucasian were <6%, <7%, 15.7%, and 73.7%, respectively; proportions of each racial category in the study population were 2.2%, 7.4%, 9.9%, and 80.5%, respectively. Incidence rates per 1000 for these were 5.95, 2.92, 5.19, and 3.01, respectively. After adjusting for age, sex, and income, race was not a statistically significant predictor. Odds of a discoid meniscus diagnosis decreased by 6% for each increment in age (p <0.001) and by 40% if male (p <0.001) in our total study population. In patients <=20 years old, sex was not a significant risk factor.

Conclusions

Younger age and female sex were identified as significant predictors for symptomatic discoid meniscus in the total study population. Unlike prior studies, this investigation did not show a significant association between this condition and race in the US, potentially increasing the diagnostic accuracy and estimated pretest probabilities for this condition based on patient demographics.

What this study adds to existing knowledge

This study provides new data on the role racial category plays in estimating the risk of having a symptomatic discoid meniscus requiring arthroscopic management, finding that it is unlikely to be a significant factor when controlling for other demographic variables. Furthermore, we report incidence statistics for this pathology in Black and Latinx populations, which so far have had little representation in peer-reviewed published literature on discoid meniscus epidemiology. In addition, this study suggests that age and sex possess statistically significant associations with a diagnosis of discoid meniscus requiring arthroscopic management, with the risk of diagnosis decreasing with age and increasing if female.

## Introduction

A discoid meniscus is a dysmorphic meniscus found to be thicker, abnormal in shape, less stable and with decreased and disorganized collagen fibers [1] compared to an anatomically normal meniscus. Due to these anatomic differences, a discoid meniscus confers inferior mechanical properties and increased risk of both meniscal tear [2,3] and symptoms such as knee pain and “snapping knee syndrome” [4]. Theories as to the development of this structural anomaly range from Smillie’s theory of congenital origins [5] to Kaplan’s refutation and suggestion that poor posterior attachment leads to aberrant meniscal hypertrophy [6]. Discoid menisci can often be discovered incidentally but also include symptomatic presentations of knee pain, “snapping” or “giving way” sensations, swelling, or locking. Past studies indicate varying accuracy of clinical assessment and limited predictive values of physical exam findings [4,7], resulting in further diagnosis requiring imaging and arthroscopy.

With any pathology, a well-developed epidemiological picture aids clinical assessment in estimating pretest probabilities and assigning an index of suspicion based on patient demographics. The aforementioned range of both etiologic explanations and clinical presentations further underscores this role of epidemiology in supporting our understanding of this pathology. Consequently, several studies have been conducted to estimate the incidence and ascertain demographic associations [5,7-14]. Of specific interest has been the incidence of discoid menisci stratified by racial or ethnic group, as several studies have reported or referred to the conclusion that the discoid meniscus has a higher incidence in Asian populations than Caucasian or other studied population groups [7,9,15]. While this association suggests a possible risk factor and observation to aid diagnoses, this has been established by relatively small, single-site studies conducted in a given country and then compared to similarly conducted studies in other countries. This had led to wide ranges of incidences being reported, such as 0.4%-20% overall incidence [8,11,13,14], 0.4%-5% [7] in Caucasian populations specifically, and 9.1%-16.6% [7] in Asian populations specifically. Rates per country include 1.8% in a Greek population [7], 16.6% in a Japanese population [11], 10.% in a Korean population [9], and 3.2 per 100,000 person-years in a US population [8]. According to one such study by Sabbag et al., single-site studies on this topic may be vulnerable to selection bias and overrepresentation [8]. Different healthcare centers, systems, and sets of guidelines may contribute to the varying incidence rates among study sites and among countries as discoid menisci--with their diverse presentations and level of symptomology--are diagnosed more or less aggressively. Furthermore, to our knowledge, there has been a paucity of research on the incidence of discoid menisci for Black and Latinx populations in the United States [16]. Given the existing corpus of studies comparing incidence per racial or ethnic group, the lack of inclusion of these groups in this discussion can problematically cause practitioners and systems to overlook patients who may be suffering from this condition, as they consider other racial/ethnic groups at higher risk by default due to availability bias of these incidence rates and lack of comprehensive analysis.

Thus, the purpose of this study was to evaluate the demographics of discoid meniscus patients with a large-scale, national database. More specifically, this investigation sought to use such a dataset to: (1) test if, in the United States, the previously reported trend of Asian populations having a higher incidence of discoid menisci is statistically identified and (2) elucidate estimates of the incidence of discoid menisci for racial/ethnic groups that have been underrepresented in studies on this condition, specifically Black and Latinx populations. Our hypothesis was that certain racial/ethnic groups would exhibit a higher incidence of this condition.

## Materials and methods

Dataset background

The dataset used Optum’s de-identified Clinformatics® Data Mart Database [17], a de-identified database consisting of commercial and Medicare Advantage claims or beneficiaries of commercial and Medicare Advantage plans. The database includes approximately 17-19 million annual covered lives, for a total of over 57 million unique lives over a nine-year period (1/2007 through 12/2017) spanning all 50 states. Data access for this project was provided by the Stanford Center for Population Health Sciences, supported by a National Institutes of Health National Center for Advancing Translational Science Clinical and Translational Science Award (UL1 TR001085) and internal Stanford Center for Population Health Sciences funding. Race and socioeconomic data were provided by an external vendor that collected data from sources such as self-reported surveys and public records. This database was used for its multi-year, nationwide scope as well as the inclusion of key demographic variables such as sex, race, and income to enable a comprehensive demographic analysis of the US population for this condition and answer our aforementioned study objectives.

Patient data were extracted using Current Procedural Terminology (CPT) codes for knee arthroscopies with meniscectomies or with meniscus repair (29880, 29881, 29882, 29883) and International Classification of Diseases (ICD-10) diagnosis codes for discoid meniscus (Q68.6). This was done to model other single-site studies’ method of study population formation [13], to define a group of cases and controls that would have presented symptomatically with meniscal pathology, and to control for potential confounding factors such as minimum level of access to healthcare required to obtain diagnostic work-up for knee pathologies including discoid meniscus. No ICD-9 code was specific for discoid meniscus; thus, the use of ICD-10 codes defined the time period considered in this study to be the interval from September 2015 (when the Optum dataset began using ICD-10 codes) to 2017 (endpoint of the available dataset). Demographic data analyzed for these patients included sex (female, male), racial category (“Asian”, “Black”, “Hispanic”, “White”), and income (reported as brackets of <$39,999, $40,000-49,999, $50,000-59,999, $60,000-74,999, $75,000-99,999, $>100,000). (Note: the dataset uses the terms “gender” to refer to binary options of male and female and “Hispanic” as a racial category. In recognition that these terms can be antiquated and inaccurate, we will refer to “female” and “male” as the patients’ biological sex and racial category as “Latinx” in our own discourse in the Introduction and Discussion, while maintaining the terms the dataset uses in the Results for the sake of congruency). Income was especially important to include, not only to assess if significantly associated with discoid meniscus diagnosis but also to factor in and control for this possible confounder in our subsequent regression model. Thus, filtering by the aforementioned CPT codes and the presence of race data formed our study population.

Analysis and statistical methods

Cases were defined as patients in the study population given a discoid meniscus diagnosis any time pre-operatively to one of the aforementioned CPT-delineated procedures and up to one-year post-operatively to allow for a delay in changing of the coding to reflect the presence of a discoid meniscus. The control population was defined as patients receiving one of these procedures who were not diagnosed with a discoid meniscus. Demographic differences between these groups were analyzed using independent samples t-test and chi-square tests. The incidence proportion of discoid meniscus was calculated for each racial category using all arthroscopy patients of the same race as the population as the denominator. Then, multivariable logistic regression models were used to evaluate the effects of race on the odds of having a discoid meniscus after adjusting for age, sex, and income level categories. Pairwise comparisons between racial groups were performed with Tukey-adjusted p-values to test for significant differences in the odds of discoid meniscus conferred by one race compared to every other. Similar pairwise analysis was conducted for income brackets. This analysis was repeated for the subset of the study population with age less than or equal to 20 years old to partially control for age as a confounder and to also provide a pediatric-specific perspective. All analyses were completed in RStudio (Boston, MA) using a two-sided level of significance of 0.05. As analyses of race often include datasets with missing data on patient race (calculated for the total available Optum dataset at 13.3%), the method of multiple imputation was attempted to address this limitation. However, the test model used to evaluate the multiple imputation demonstrated low accuracy of imputing the missing race data and this approach was deemed not appropriate to use by the study group's statisticians.

## Results

In the time period of September 2015 (when the Optum dataset began using ICD-10 codes) to 2017 (endpoint of the available dataset), there were 1006 discoid meniscus cases, 776 of which had race data. Of these, 198 cases underwent one of the aforementioned arthroscopic procedures. In total during this timeframe, 60,042 meniscectomy or meniscal repair arthroscopic procedures with race data were recorded. This led to an incidence proportion of 0.309% or 3.09 in 1000.

Demographic differences

Breakdown by reported racial group of our study population can be seen in Table 1 and Table 2. Race and discoid meniscus diagnosis presence are significantly associated (p=0.016), but post-hoc tests with Bonferroni adjustments yielded non-significant results (adjusted p-value > 0.05) within each race. The one exception was for the Hispanic population (adjusted p-value = 0.039), indicating this population had a proportion of cases of discoid meniscus requiring arthroscopic management statistically higher compared to its proportion of patients receiving arthroscopies with meniscectomy or meniscal repair not due to having a discoid meniscus. Regarding age, the median age at discoid meniscus diagnosis was 29 years old (interquartile range of 16-51 years), compared to a median of 56 years old (interquartile range of 45-65 years) for the patients receiving arthroscopies with meniscectomy or meniscal repair not due to having a discoid meniscus. This difference was also statistically significant (p < 0.001). Regarding sex, 56.6% of discoid meniscus patients were female, a statistically larger proportion of the discoid meniscus population compared to the proportion of female controls in the study population. Income levels were not significantly associated with a discoid meniscus diagnosis. 

**Table 1:** Demographic breakdown of study population based on race and sex. *Note: exact number withheld due to dataset requirements for small cell sizes to preserve de-identification of patients. Precise values were used in all statistical testing and conclusions. Bold values represent statistically significant.

	Discoid meniscus requiring arthroscopic management (n = 198)		Arthroscopic procedure but no discoid meniscus (n = 60,042)				
	Count	%	Count	%	Population-level incidence (per 1000)	p-value	Bonferroni adj. p-value
Race							
Asian	<11*	<6%*	1,337	2.2%	5.95	0.016	0.356
Black	<15*	<7%*	4,440	7.4%	2.92		>0.999
Hispanic	31	15.7%	5,945	9.9%	5.19		0.039
White	146	73.7%	48,320	80.5%	3.01		0.086
Sex							
Female	112	56.60%	28,965	48.20%	3.85	0.023	-
Male	86	43.40%	31,077	51.80%	2.76		

**Table 2:** Demographic breakdown of study population based on age. Bold values represent statistically significant.

	Discoid meniscus requiring arthroscopic management (n = 198)		Arthroscopic procedure but no discoid meniscus (n = 60,042)		
	Median (years)	Interquartile range	Median (years)	Interquartile range	p-value
Age	29	16-51	56	45-65	<0.001

Upon analyzing the subset of the study population with age less than or equal to 20 years, similar results were found for race as seen in Table 3 - no racial group and no income bracket had a significant association. Of note, in this subset, there was not a significant association for sex with diagnosis of a discoid meniscus requiring arthroscopic management. Only age was significantly different between the discoid meniscus group and the broader study population, with the median age at diagnosis was 15 years old (interquartile range of 12-17 years) while the median age was 17 years old (interquartile range of 16-18 years) of the patients receiving arthroscopies with meniscectomy or meniscal repair but not due to having a discoid meniscus.

**Table 3:** For subset of age <= 20 years, demographic breakdown of study population based on race and sex. *Note: exact number withheld due to dataset requirements for small cell sizes to preserve de-identification of patients. Precise values were used in all statistical testing and conclusions.

	Discoid meniscus requiring arthroscopic management (n = 79)		Arthroscopic procedure but no discoid meniscus (n = 3905)			
	Count	%	Count	%	Population-level incidence (per 1000)	p-value
Race						
Asian	<11*	<14%*	112	2.8%	34.48	0.271
Black	<11*	<14%*	284	7.1%	20.69	
Hispanic	13	16.5%	480	12.0%	26.37	
White	56	70.9%	3,109	78.0%	17.69	
Sex						
Female	42	53.2%	1,728	43.4%	23.73	0.104
Male	37	46.8%	2,257	56.6%	16.13	

Multivariable regression

As reflected in Table 4, the results of the logistic regression model using these demographic variables to predict for discoid meniscus diagnosis status revealed that the odds of having discoid meniscus requiring arthroscopic management among our study population did not significantly differ by race (p > 0.05 for all), once adjusting for other demographic characteristics and considering all pairwise comparisons between racial groups with Tukey adjusted pairwise comparisons. Age and sex are both significant predictors, with the odds of discoid meniscus decreasing by 6% for every increase of 1 year in age (p < 0.001) and the odds of discoid meniscus being 43% lower for male patients (p < 0.001) as compared to female patients. None of the compared pairs of income brackets resulted in significant Tukey adjusted pairwise p-values.

**Table 4:** Odds ratios and significance of covariates of logistic regression. *Odds ratio is with respect to subpopulation of patients who are Asian; Tukey adjusted pairwise p-values also generated for all pairwise comparisons between racial groups, with none meeting threshold of significance.

	Odds ratio	Lower 95%	Upper 95%	p-value
Race (Black)*	0.56	0.23	1.43	0.201
Race (Hispanic)*	0.91	0.43	2.15	0.815
Race (White)*	0.68	0.35	1.51	0.287
Age	0.94	0.93	0.94	<0.001
Sex (Male)	0.57	0.43	0.75	<0.001
Income ($40K-$49K)	0.55	0.22	1.18	0.158
Income ($50K-$59K)	1.21	0.65	2.15	0.535
Income ($60K-$74K)	0.68	0.36	1.24	0.227
Income ($75K-$99K)	0.77	0.47	1.28	0.319
Income (>$100K)	0.70	0.47	1.07	0.089

For the $\leq$ 20 years old subset, the model produced a similar result regarding race (p > 0.05 for all), as seen in Table 5. Age continued to be a significant predictor, with the odds of discoid meniscus decreasing by 34% for every increase of one year in age (p < 0.001). However, sex was not a significant predictor in this subset. Income continued to not be a significant predictor.

**Table 5:** For subset of age <= 20 years, odds ratios and significance of covariates of logistic regression. *Odds ratio is with respect to subpopulation of patients who are Asian; Tukey adjusted pairwise p-values also generated for all pairwise comparisons between racial groups, with none meeting threshold of significance.

	Odds ratio	Lower 95%	Upper 95%	p-value
Race (Black)*	0.64	0.17	2.73	0.519
Race (Hispanic)*	0.70	0.22	2.69	0.566
Race (White)*	0.47	0.18	1.64	0.173
Age	0.68	0.63	0.73	<0.001
Sex (Male)	0.87	0.54	1.40	0.561
Income ($40K-$49K)	1.34	0.36	4.19	0.631
Income ($50K-$59K)	1.07	0.32	3.21	0.903
Income ($60K-$74K)	0.25	0.04	1.00	0.084
Income ($75K-$99K)	0.59	0.22	1.55	0.281
Income (>$100K)	0.70	0.35	1.55	0.345

## Discussion

In our study population, age and sex possess statistically significant associations with diagnosis of a symptomatic discoid meniscus requiring arthroscopic management, with the risk of diagnosis decreasing with increasing age and increasing if female. Asian and Latinx populations did report incidences approximately twice that of other racial groups, but no racial category possessed a statistically significant association in our study population once controlling for other demographic variables.

The current corpus of research often compares the incidence statistics produced by single-site studies in various countries across the globe, finding that incidence rates reported within Asian populations are often higher than that of Caucasian populations [7,9,15]. Regarding specific incidence calculations, reported ranges have been quite broad, with often cited statistics including 0.4%-20% generally [8], 0.4%-5% [7] in Caucasian populations specifically, and 9.1%-16.6% [7] in Asian populations specifically. The widths of these ranges could be due to these studies’ sample sizes, lack of controlling for confounding demographic factors, and the heterogenous definitions of study populations used, which range from the general population of a county, to available cadaveric knees, to patients receiving arthroscopies for meniscal pathologies. While the calculated incidences in this study initially support the finding of Asian populations having a higher incidence rate than Caucasian or other racial groups, application of statistical testing and controlling for other demographic factors such as age, sex, and income suggests the conclusion that it is unlikely that being a member of any racial category actually increases a patient’s odds of having a discoid meniscus requiring arthroscopic management. Of note, it appears that this conclusion is supported when analyzing a broader, more diverse population rather than a single site, as demonstrated by Grimm et al.’s study of a large Southern Californian population [16]. If further research confirms such findings, this conclusion could reduce potential clinical bias that overestimates the importance of the association between discoid meniscus pathology and race.

Regarding age, the current study suggests that increasing age provides a decreasing risk of diagnosis of a discoid meniscus that required arthroscopic management, consistent with the findings of past works [2,13]. The median age at diagnosis reported in this study (29 years) is consistent with age statistics reported in some past research. Papadopoulos et al. reported mean age of discoid meniscus cases of 31 years old [7] and Kim et al. reported the highest incidence in the 21- to 30-year-old age group [9]. However, both younger and older median ages have been reported in past studies, with Nathan and Cole reporting a mean age of 22 years [18] and Dickhaut and DeLee describing an older cohort presenting in the fourth decade [12,13]. Overall, it appears that the preponderance of the literature supports the findings of a relatively young age of diagnosis [13]. Further, the broader observed relationship of age being inversely related to risk of discoid meniscus diagnosis appears to be consistent with existing studies.

Regarding sex, this study found that female patients in the total study population have an increased risk of a symptomatic discoid meniscus requiring arthroscopic management, but that the subset of patients of age $\leq$ 20 years did not demonstrate this association. While some past studies also propose a relationship between sex and risk of discoid meniscus [9], several others have reported similar incidence or no significant difference in incidence between sexes [7,8,16,18]. Speculatively, the age-dependent relationship described in this study could imply that the effect of sex as a risk factor is delayed or accumulates over time. Interestingly, Ikeuchi et al. reported female patients have a younger average age of presentation [11], suggesting an interplay between sex and age. Of note, the extent to which age, sex, and other demographic variables are controlled for is quite variable across these single-site studies. Further work in a dataset with a more robust set of demographic variables is needed to clarify the age and sex as risk factors.

The limitations of this study are largely related to those inherent in using a dataset such as the one used for this study. While its national scope and range of demographic data collected are useful aspects that made this study’s analysis possible, claims-based databases often identify diagnoses and procedures based on ICD and CPT codes, as in this study. Given the codes available, this study only provides analysis for symptomatic discoid meniscus requiring surgical management. Compared to medical records and imaging results available in a chart review-based study, these codes also provide data that is not as high-dimensional and granular. Future work with more in-depth clinical information could study a potentially insightful study population of asymptomatic, MRI-diagnosed discoid menisci or discoid menisci managed non-surgically.

One obstacle our analysis needed to account for relates to missing patient race data in the dataset, limiting the sample size and potentially limiting accuracy if this data was missing in a non-random fashion. As aforementioned, an attempt was made to address this limitation through multiple imputation, as demonstrated in a similar analysis [19]. However, this was met with unsatisfactory results and risk of introducing bias. Thus, our solution was to have the presence of race data as an essential component of our inclusion criteria.

## Conclusions

Due to the range of symptomatic presentations and differential diagnoses for potential etiologies, understanding the epidemiology of the discoid meniscus can be useful in aiding our understanding and clinical assessment of this condition. This study provides new data on the role racial category plays in estimating the risk of having a symptomatic discoid meniscus requiring arthroscopic management, finding that it is unlikely to be a significant factor when controlling for other demographic variables. Furthermore, we report incidence statistics for this pathology in Black and Latinx populations, which so far have had little representation in peer-reviewed published literature on discoid meniscus epidemiology. In addition, this study suggests that age and sex possess statistically significant associations with a diagnosis of discoid meniscus requiring arthroscopic management, with the risk of diagnosis decreasing with age and increasing if female.
